# Topical Administration of Ibuprofen for Injured Athletes: Considerations, Formulations, and Comparison to Oral Delivery

**DOI:** 10.1186/s40798-017-0103-2

**Published:** 2017-10-05

**Authors:** Martin Anthony Christopher Manoukian, Christopher William Migdal, Amode Ravindra Tembhekar, Jerad Alexander Harris, Charles DeMesa

**Affiliations:** 1 University of California Davis School of Medicine, 4610 X Street, Sacramento, CA USA; 2 Department of Anesthesiology and Pain Medicine, University of California Davis School of Medicine, 4610 X Street, Sacramento, CA USA

## Abstract

Non-steroidal anti-inflammatory drugs (NSAIDs) are a class of drugs commonly used to treat both the acute and chronic injuries sustained by athletes during training and competition. In many parts of the world, NSAIDs can be purchased over-the-counter and used without any physician oversight. However, the chronic nature of overuse injuries requires NSAIDs to be taken orally for an extended period of time. As a result, they can have significant adverse effects on athletes, namely gastrointestinal (GI), renal, and cardiovascular damage. Dyspepsia and upper GI ulceration and bleeding are of great concern in chronic NSAID use, and as such oral NSAIDs are generally contraindicated in those with a history of peptic ulcers or irritable bowel disease. In the setting of chronic overuse soft tissue or joint disease, topically administered NSAIDs offer an alternate route of administration that has the potential to deliver a similar level of pain and anti-inflammatory relief while bypassing the harmful side effects associated with oral intake. Topically applied NSAIDs are able to achieve high concentrations within the targeted site of action while simultaneously keeping plasma concentrations low, offering several advantages over oral administration. One commonly used generic NSAID is ibuprofen (2-(4-isobutylphenyl)propanoic acid). First synthesized in the 1960s, ibuprofen has since become widely available as an over-the-counter pharmaceutical. In this review, we outline new and different techniques that have been used to deliver ibuprofen into diseased tissues, including supersaturations, microemulsions, gels, nanosystems, and microneedles. We also review relevant clinical trials comparing transdermally delivered ibuprofen to placebo and orally administered ibuprofen.

## Key Points

- Ibuprofen’s diffusion and absorption characteristics make it an optimal therapeutic for transdermal delivery to affected tissue in injured and recovering athletes.
- Topically administered ibuprofen has been shown to be superior to placebo in the treatment of joint and soft tissue injury.
- Topically administered ibuprofen has been shown to be equally effective to orally administered ibuprofen in the treatment of joint and soft tissue injury and is associated with a lower incidence of unwanted gastrointestinal side effects.

## Review

### Introduction

Non-steroidal anti-inflammatory drugs (NSAIDs) are among the most commonly prescribed drugs in the world [1–3]. While available under many brand names and formulations, NSAIDs share a common mechanism of action through the inhibition of cyclooxygenase 1 and cyclooxygenase 2 (COX2). The primary anti-inflammatory effects of NSAIDs are rooted in the inhibition of COX2-mediated oxygenation of arachidonic acid, a key step in the synthesis of inflammatory prostaglandins [4, 5]. Indications for NSAID use include osteoarthritis (OA), soft tissue injury, and rheumatoid disease, among others [1, 2, 5–7]. These indications are of particular concern for athletes, especially elite athletes, who suffer higher rates of soft tissue injury than non-athletes and are at a greater risk of developing OA [8–12]. For the injured athlete, studies have indicated that NSAID therapy can increase the rate of muscle recovery via modulation of the inflammatory response to muscle injury [13, 14]. In addition to attenuating the inflammatory response, the analgesic effect of NSAIDs may assist in pain management in the recovering athlete, allowing for increased tolerance of rehabilitation exercises [15]. This can reduce potentially detrimental consequences of muscle deconditioning secondary to immobilization [15, 16].

Although NSAIDs have demonstrated many therapeutic benefits and have a strong safety profile allowing for over-the-counter availability in the USA, they are not without adverse effects. Of those treated with chronic oral NSAID therapy, 90% are at risk of adverse gastrointestinal (GI) effects, with the annual incidence of upper GI ulcers ranging from 2 to 4% [17–20]. Furthermore, patients treated with oral NSAIDs have been shown to have an elevated risk of cardiovascular events and increased incidence of heart failure [21]. Two studies by Lanas et al. concluded that of patients treated with oral NSAIDs for OA, 90% were at risk for GI adverse effects and 44.3% were at risk for adverse cardiovascular effects [17, 20]. In addition, NSAIDs have been shown to reduce the cardiovascular protective benefits of low-dose aspirin when taken simultaneously [21–24]. Furthermore, renal toxicity is also a concern in patients who use NSAIDs [25–27]. Altogether, the use of NSAIDs has been estimated account for 107,000 hospitalizations and 16,500 deaths annually in the USA alone [28].

Studies of the adverse effects of NSAID therapy have primarily focused on oral NSAIDs, with the increased risk of adverse effects resulting from high plasma concentrations. To avoid such adverse side effects, studies have looked at both modifications of drug action and alternative methods of drug delivery. Celecoxib was introduced as a COX2 selective inhibitor that avoided the adverse GI side effects of oral non-selective NSAIDs [29]. However, a risk of cardiovascular events was found to be equivalent to that of traditional NSAIDs [30–32]. Other attempts have been made to alter the chemical structure of ibuprofen to orally deliver the same desired therapeutic effects without the undesired ulcerogenic effects [33]. Perhaps most promising, studies have suggested that topical application of NSAIDs via gels, creams, sprays, or plasters are as effective as oral NSAIDs and yet are associated with fewer adverse side effects [1, 34–36]. This review focuses on the topical delivery of ibuprofen, 2-(4-isobutylphenyl)propanoic acid, a widely used NSAID. Topical ibuprofen’s effectiveness at treating chronic musculoskeletal pain is comparable to other topical agents such as diclofenac and ketoprofen [1]. However, no overview of the current state of research in topical ibuprofen is currently available. In this article, we will highlight the key technical aspects of transdermal pharmaceutical delivery, review research in novel formulations used as delivery vehicles, compare the efficacy of topical ibuprofen against placebo and oral ibuprofen, and touch upon the costs associated with topical ibuprofen administration for the injured athlete.

### Skin Barrier

**Fig. 1:**
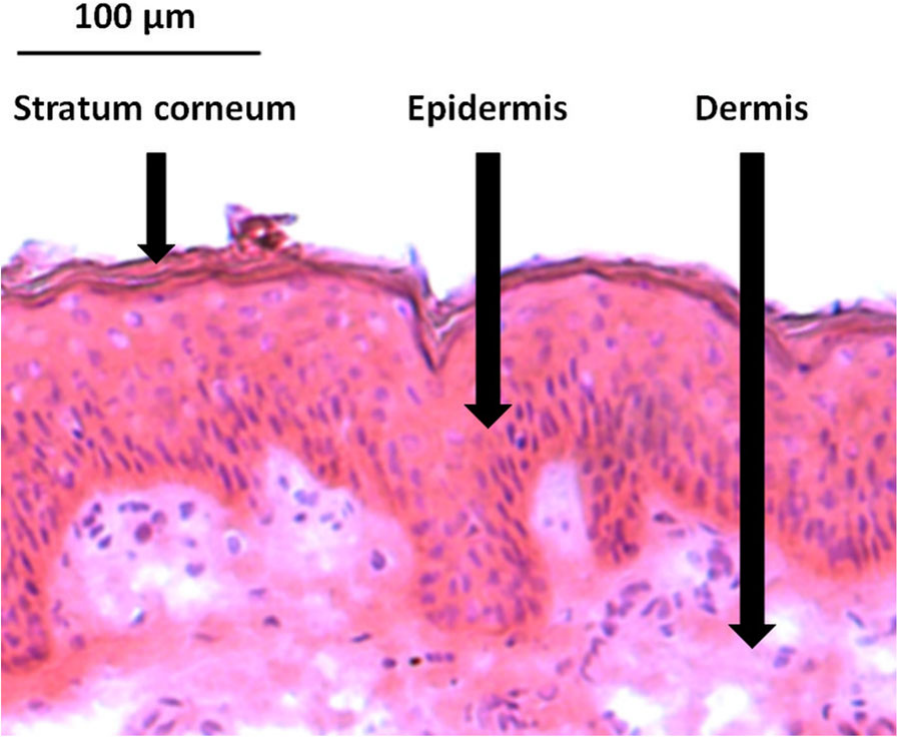
Hematoxylin and eosin staining of human skin, illustrating the layers of the stratum corneum, epidermis, and dermis. Image provided by author MACM

The skin is made up of two functional layers, the epidermis and the dermis (Fig. 1). The role of the epidermis is to protect the body from foreign materials and mechanical trauma as well as prevent loss of water. The superficial layer of the epidermis is the stratum corneum, a 10–20-μm thick layer of terminally differentiated keratinocytes known as corneocytes. In addition to being covered by a lipid film, the stratum corneum also has numerous intercellular lipids consisting of ceramids, cholesterol, and saturated free fatty acids [37]. Due to this intensely lipid-rich character, the stratum corneum is largely impermeable to hydrophilic substances, posing a hurdle to any drug that is to be delivered topically. Below the stratum corneum sits the remainder of the epidermis, which is devoid of vasculature, and instead receives all of its nutrients via diffusion from the dermis.

Below the epidermis sits the dermal-epidermal junction, also known as the basement membrane zone. Made up of hemidesmosomes, laminins, collagens, and glycoproteins, the basement membrane zone’s main function is to keep the epidermis attached to the dermis and withstand external shearing forces. Like the glomerular basement membrane, the dermo-epidermal basement membrane is charged, imparting selectivity to diffusion. However, the charge of the dermo-epidermal basement membrane is far less than that found in the glomerulus [38, 39]. Thus, although it does impart some selectivity to absorption, the dermo-epidermal basement membrane is much more permeable to charged molecules than the glomerular basement membrane.

The deepest layer of the skin is the collagenous dermis. Made up mostly of type I collagen, the dermis serves to add pliability and flexibility to the skin, while also serving an important role in thermoregulation of the body and housing the various skin appendages. These appendages, which include hair, sebaceous glands, and eccrine and apocrine sweat glands, can serve as conduits of drug delivery and absorption into the dermis [40]. Importantly, and in contrast to the epidermis, the dermis is heavily vascularized [41]. Thus, any drug that diffuses into the dermis may diffuse into blood vessels to be transported downstream and eventually reach systemic circulation. This must be taken into account with drugs that are potentially toxic if they are carried to parts of the body other than their intended sites of action.

#### Traversing the Skin Barrier

Two routes of topical transdermal delivery include traveling through the epidermis itself or, alternatively, through the skin appendages. Because of their large size, skin appendages allow for the absorption of larger molecules and are much less selective [37]. However, disadvantages of this approach include excessive sebum within the follicle, sequestration of drug within the follicle, and lack of appendages in some areas of the body [40]. Thus, trans-epidermal routes must also be considered.

Direct delivery of drugs through the epidermis is complicated by its intensely lipophilic character. While this is important in preventing water from escaping the body, it also makes transdermal delivery of hydrophilic drugs nearly impossible. An often cited rule is the Rule of 5 proposed by Lipinski et al. which states that non-oral delivery of drugs can only be accomplished if the drug has a molecular weight below 500 Da, has less than five H-bond donors, has less than ten H-bond acceptors, and has an octanol-water partition coefficient (also known as log P, which is a measure of lipophilicity) below five [42]. Furthermore, a follow-up paper by Choy and Prausnitz suggests that transdermal drug delivery is especially difficult when compared to drugs crossing the ophthalmic or pulmonary epithelium [43]. This must be taken into consideration when preparing a vehicle with which to deliver ibuprofen. However, the physical characteristics of ibuprofen all fall well within Lipinski’s criteria, favoring its use in transdermal delivery systems (Table 1) [44]. This allows ibuprofen to efficiently diffuse through the epidermal and dermal layers into the soft tissue and connective tissue at its point of action in a way equal or even superior to orally administered ibuprofen [45, 46].

**Table 1:** Comparison of physical characteristics of select NSAIDs to Lipinski’s Rule of 5 [42, 44, 103–105]

	Lipinski’s Rule of 5	Ibuprofen	Ketoprofen	Diclofenac	Celecoxib
Molecular weight, (Da)	< 500	206	254	296	381
H-bond donors	< 5	1	1	2	1
H-bond acceptors	< 10	2	3	3	7
Log P	< 5	3.97	3.1	4.4	3.4

It should be noted, however, that there are many factors specific to the athlete that can cause variation in the transdermal diffusion and delivery of therapeutics. Sex, age, ethnicity, skin hydration, skin temperature, obesity, disease state, and anatomical site of application all play a role in determining the diffusion rate of therapeutics through the skin [47, 48]. Application to sites with a thick epidermis, such as the palms or soles of the feet, for example, makes it more difficult for drugs to penetrate than the skin of the arm or trunk.

#### Into the Synovium

For more efficient relief of joint swelling and pain in intra-articular disease, transdermally delivered ibuprofen must penetrate into the synovium. All joints in the body are composed of a fibrous outer capsule that connects the articulating bones involved. Within this outer capsule lies the inner synovial membrane which is filled with synovial fluid. This fluid utilizes hyaluronic acid and lubricin to protect the joint from damage inducing friction. Over time, repeated stress and injury to the joints leads to a breakdown of the synovial capsule and articular cartilage. This then leads to the increased production of pro-inflammatory cytokines, synovitis, chondritis, and pain [49]. To be effective, transdermally delivered ibuprofen must be able to diffuse into the synovium itself to counteract the inflammation and provide the patient with symptomatic relief.

To get into the synovial joints, transdermally delivered ibuprofen must either diffuse directly through the articular capsule, or it must first diffuse into the capillaries of the dermal papillae and then be carried by the bloodstream until it is deposited within the synovial fluid. Once within the synovial fluid, ibuprofen can act on both the synoviocytes and the chondrocytes of the articular cartilage, which obtain most of their nutrients via diffusion from the synovial fluid [50]. Ibuprofen reduces intra-articular inflammation by decreasing the expression of pro-inflammatory cytokines, including matrix metalloproteinase 3, matrix metalloproteinase 13, interleukin 6, and tumor necrosis factor alpha [51].

#### Difference in Enantiomer Selection

Importantly, it has been shown that the S-enantiomer of ibuprofen has a greater ability to diffuse into and stay localized within the synovial joint than the R-enantiomer. This is believed to be due to the higher serum protein binding affinity of the R-enantiomer [52]. Additionally, the S-enantiomer of ibuprofen has been shown to have low cytotoxicity to both chondrocytes and synoviocytes, making it an excellent candidate for use in transdermal delivery, particularly for OA [53].

### Formulation Techniques for Transdermal Ibuprofen Delivery

Multiple commercially available formulations of topical ibuprofen exist, with most containing 5% by weight of ibuprofen. However, despite the same concentration of the drug, different products often exhibit significantly variable uptake and delivery through the skin [54]. Thus, current areas of research include the development and testing of superior formulations of transdermal ibuprofen via a variety of drug delivery vehicles. Supersaturations, microemulsions, nanosystems, and microneedles are a few of the delivery mechanisms tested by researchers in the search for the optimal transdermal delivery vehicle.

#### Supersaturations

Supersaturated solutions of ibuprofen with disodium hydrogen phosphate allow for higher concentrations of ibuprofen in solution than would be possible under normal circumstances. These have previously been shown to greatly increase flux across the human epidermis, indicating that they can disrupt and penetrate through the stratum corneum [55]. Similar results were achieved using the cellulosic polymer hydroxypropyl methylcellulose [56]. Moreover, the combination of ibuprofen sodium and polymer hydroxypropyl methylcellulose was shown to inhibit nucleation and crystal growth, prolonging drug supersaturation [57]. Despite these advances, long-term stability of supersaturated solutions remains an area of concern and active research.

#### Microemulsions

Microemulsions (systems of oil, water, and an amphiphilic compound) have similarly been shown to allow for high concentrations of ibuprofen in solution (up to 3%). Ethyl oleate, in particular, allows for greater permeability and solubility in skin [58]. A later study also found that ibuprofen-loaded microemulsions released comparable amounts of the drug in vitro to a commercial ibuprofen hydrogel, demonstrating their viability as a drug delivery vehicle [59]. In an experiment measuring the effect of saturated fatty acid length on the permeability of ibuprofen transdermal microemulsions, medium-length fatty acid-based microemulsions had low toxicity, good permeability, and the highest analgesic activity [60]. One group described an optimum formulation of a microemulsion consisting of 6% oleic acid, 30% Cremophor RH40/Transcutol (Xietai Chemical Co. Ltd., Shanghai, China/Gattefosse, Shanghai, China) P (2:1, *w*/*w*), and 59% water phase that showed high permeation with no signs of skin irritation [61].

#### Gels

Ibuprofen gels are commercially available and used in clinical practice, but efforts continue to improve skin permeability, bioavailability, and safety. An optimized transdermal gel consisting of 5% ibuprofen, 30% ethanol, and 10% POE [5]cetyl/oleyl ether had significantly greater bioavailability than the two commercial gels Ibutop® (Deutsche Chefaro Pharma GmbH, Germany) and Senterlan® (Unicorn Laboratories, Hong Kong) [62]. Oxidized cellulose-based gels also exhibited similar in vivo stratum corneum uptake and skin penetration compared to over-the-counter marketed formulations, despite 80% less drug loading [63].

#### Nanosystems

While novel formulations of gels have been shown to be superior to commercial products, even more promising is the incorporation of nanosystems into transdermal ibuprofen gel. Nanosystems allow for the production of gels designed at the atomic and molecular level. For example, an ethosomal ibuprofen gel containing 200 nm unilamellar vesicles was applied transdermally in rats and was found to have an efficient analgesic effect that lasted at least 6 h with no evidence of skin irritation [64]. Another gel using a nanostructured lipid carrier improved diffusion through the epidermis compared to traditional ibuprofen gel formulation by increasing the lipophilicity of ibuprofen [65]. An ibuprofen nanoliposome preparation containing phosphatidylcholine, cholesterol, and dicetyl phosphate also showed superior skin permeation in in vitro and in vivo experiments using human skin [66].

#### Microneedles

Dissolvable microneedles can cross the stratum corneum without damaging dermal nerves and blood vessels in order to deliver drugs in a similar manner to transdermal patches. They have repeatedly been demonstrated to be an efficient way of directly introducing pharmaceuticals into the dermis, including small molecules, vaccines, and even large proteins [67–70]. Similarly, ibuprofen-laden dissolvable microneedles have been shown to deliver local ibuprofen sodium effectively into rats [71]. However, these experiments suggest that high plasma concentrations may be achieved in the process, negating the safety advantages of transdermal ibuprofen over oral delivery. In addition, absolute sterilization of microneedles (which would ultimately be required by regulatory bodies such as the US Food and Drug Administration and the European Medicines Agency) cannot be achieved by conventional methods without damaging the vehicles. Gamma radiation has been explored as a possible sterilization technique, yet even this elicits an altered permeation profile of ibuprofen-laden microneedles [72]. Greater optimization of microneedles is needed to produce sterile and effective products with minimal risk for toxicity.

#### Summary

Overall, novel formulations of transdermal ibuprofen using diverse delivery methods have been demonstrated to be safe and more efficacious than commercial preparations in studies using both animal models and the human epidermis. Optimum formulations using these techniques should be incorporated into clinical trials comparing the effectiveness of transdermal ibuprofen to traditional methods of delivery.

### Efficacy

#### Topical Ibuprofen vs Placebo and Non-Pharmacological Agents

Topical ibuprofen has been demonstrated to be superior to placebo in treating OA of the knee acutely [45, 73, 74]. These studies only focused on short-term outcomes of 1–2 weeks, yet they demonstrated improved Western Ontario and McMaster Universities Arthritis Index (WOMAC) total, WOMAC physical function, and visual analog scale pain scores during joint motion. Other studies have shown that continuous ultrasound and topical arnica are equally effective as topical ibuprofen in relieving pain in knee OA and hand OA at 3 weeks, respectively [75, 76]. There is a paucity of data comparing the long-term effects of topical ibuprofen and placebo in the treatment of knee OA. This would be an interesting future study, as there is some evidence suggesting that NSAIDs may not be superior to placebo in reducing pain beyond 2 weeks in patients with knee OA [77, 78].

Similar to OA, few studies have evaluated the efficacy of topical ibuprofen in treating acute soft tissue injuries. One study demonstrated a clinically meaningful reduction in pain in 75% of patients using topical ibuprofen compared to 39% of patients receiving placebo at 7 days [79]. The same study found a significant reduction in interference of physical activity in patients receiving topical ibuprofen vs placebo, 79 and 44%, respectively (*p* = 0.001). Another study concluded that topical ibuprofen is superior to placebo in reducing visual analog scale pain scores during rest, standing, and walking 48 h post-treatment in acute ankle sprains (*p* < 0.05) [80]. Based on these studies, topical ibuprofen is likely effective for treating acute soft tissue injuries, although additional research is needed to provide more clarity.

#### Topical Ibuprofen vs Oral Ibuprofen

Oral ibuprofen has been a mainstay of treatment for chronic and acute musculoskeletal pain for many years and its efficacy is well-documented. However, given that topical NSAIDs are theorized to have fewer side effects than oral NSAIDs, it is important to understand if the efficacy of topical ibuprofen is equal to the efficacy of oral ibuprofen. The Topical or Oral Ibuprofen (TOIB) study of 585 patients with chronic knee pain showed that topical and oral ibuprofen demonstrated equivalent changes global WOMAC scores at 12 months [78]. It is important to note, however, that neither treatment demonstrated a clinically or statistically significant reduction in global WOMAC scores from baseline. A smaller study of 20 patients with chronic knee pain treated with topical or oral ibuprofen demonstrated equal significant improvements at 2 weeks in WOMAC pain, stiffness, and physical function scores [81]. These studies demonstrate that topical ibuprofen is as effective as oral ibuprofen in the treatment of chronic knee pain.

In one double-blind study of 100 patients with acute soft tissue injuries, topical ibuprofen demonstrated equal time to (1) being “completely better,” (2) significant relief of pain at rest, (3) significant relief of pain while moving, and (4) resolution of swelling when compared to oral ibuprofen [82]. Every study that we examined above concluded that topical ibuprofen has comparable efficacy to oral ibuprofen, which should make it an attractive alternative in the treatment of acute and chronic musculoskeletal injuries given its favorable safety profile [78, 81, 82].

## Bioavailability and Side Effects

### Bioavailability

One key advantage of topical ibuprofen use is its decreased absorption and systemic distribution when compared to oral ibuprofen. One study showed that plasma concentrations of oral ibuprofen were 300 times that of plasma concentrations of topical ibuprofen and that 0.55% of administered topical ibuprofen is eliminated in the urine in 24 h compared to 97% of oral ibuprofen [83]. Other studies have found that topical ibuprofen has 2–8% of the peak serum concentration of the manufacturer’s reported plasma concentrations after oral administration [2, 84]. However, Kleinbloesem et al. found that the dose-corrected bioavailability of topical ibuprofen was 22% that of oral ibuprofen [85]. Though this is a much higher bioavailability than found in the previously cited studies, this still represents a fraction of the bioavailability found in oral consumption.

### Side Effects

With the exception of the TOIB study, all of the clinical trials included above in this paper reported no adverse outcomes in either the oral ibuprofen or the topical ibuprofen groups [45, 73–82]. This is not entirely surprising, as their follow-up times were all less than 3 weeks. However, the TOIB study found no significant difference in severe adverse effects requiring hospitalization between patients taking oral or topical ibuprofen [78]. However, 16% of patients changed from oral to topical ibuprofen due to adverse effects, while only 1% of patients switched from topical to oral ibuprofen due to adverse effects. In general, side effects of topical NSAIDs occur in about 10–15% of patients and are overwhelmingly cutaneous allergic reactions related to patches or other topical preparations [2, 78, 86, 87]. Nevertheless, it is important to note that because some topically applied ibuprofen is absorbed and distributed systemically, systemic side effects are still of concern.

#### Gastrointestinal Side Effects

A large review estimated that oral NSAIDs are associated with a 15% incidence of adverse GI effects, whereas adverse GI effects are very rare with topical NSAID use [2]. In a case-controlled study that examined 1100 patients admitted to the hospital for upper GI bleeding or ulceration, oral NSAIDs were strongly associated with perforation (odds ratio 4.8, *p* < 0.001) and bleeding (odds ratio 1.74, *p* < 0.001) [88]. However, topical NSAID use was not associated with upper GI perforation or bleeding, with an odds ratio of 0.86 (*p* = 0.89) for perforation and 1.05 for bleeding (*p* = 0.86). Although these previous two studies were not specific for ibuprofen, they included both topical and oral ibuprofen as parts of their NSAID aggregate.

#### Renal Side Effects

There is a scarcity of studies available that have directly investigated the renal side effects of topical NSAIDs relative to their oral counterparts. Prior studies indicate the 24-h renal excretion of topical vs oral ibuprofen is 0.57 and 97%, respectively, suggesting that topical therapy may be much safer in patients with renal impairment [83]. Furthermore, a case-control study of 207 patients hospitalized for acute renal failure concluded that there was a minimal independent risk of renal damage in those treated with topical NSAIDs [89]. However, two studies have documented cases of interstitial nephritis resulting from topical ibuprofen and topical piroxicam [90, 91]. Nevertheless, this risk was no greater than that of oral NSAIDs and is in fact much less as toxic systemic levels are rarely achieved with topical administration [92].

#### Cardiovascular Side Effects

As with renal side effects, there is little literature that explores the cardiovascular side effects of topically administered ibuprofen. Oral ibuprofen, however, has been repeatedly demonstrated to negatively affect aspirin’s ability to prevent platelet aggregation and should be avoided in athletes who are taking aspirin prophylactically to avoid adverse events such as myocardial or cerebral infarction [93–98]. Although transdermally administered ibuprofen’s decreased absorption and systemic distribution may make it a safer option than oral ibuprofen, there are few data in the literature to support its use in the setting of concomitant aspirin use, and more research in this field is required. Alternatively, NSAIDs such as celecoxib and diclofenac (both oral and transdermal) have already been shown to be safe when used concurrently with aspirin and should be considered instead [95, 96, 99].

## Costs

A major drawback in using topical NSAIDs is the increased cost associated with topical formulations. For example, a 10-day treatment with oral ibuprofen would, on average, cost about $3 for plain tablets, while a 10-day supply of diclofenac gel would cost about $65 [100]. However, it is not entirely clear that oral NSAIDs are less expensive than topical NSAIDs in patients treated chronically. A study estimated that one third of the total cost of treating patients with OA is dedicated to treating the NSAID-induced adverse GI side effects that occur in up to 25% of the patients [101]. A branch of the 2-year TOIB study ambivalently concluded that oral NSAIDs are probably less expensive when considering quality-adjusted life years, although they emphasized the importance of future research [102]. Cost is currently a large barrier to recovering athletes who wish to receive treatment with topical NSAIDs in the USA, as topical formulations are more expensive than over-the-counter oral formulations. Thus, they are typically reserved for patients who cannot tolerate oral NSAIDs or are more prone to side effects due to oral NSAIDs. However, the increased cost associated with topical ibuprofen may be partially offset by the decreased cost of treating drug-related side effects.

## Conclusion

Ibuprofen is a NSAID that is widely used by injured athletes to alleviate pain and inflammation associated soft tissue injury and OA. Although oral administration of ibuprofen is associated with numerous side effects, topically administered ibuprofen allows for drug delivery while reducing the risk of adverse GI, renal, and cardiovascular side effects. Many different formulations have been created in an attempt to increase diffusion of ibuprofen through the skin and into its site of action, including supersaturation, microemulsion, gel, nanosystem, and microneedle techniques. Transdermal ibuprofen delivery has been shown to be superior to placebo and comparable to oral ibuprofen in terms of efficacy, while also leading to fewer or less serious side effects. Topical ibuprofen may also be more cost-effective in the long term due to decreased rates of complications and hospitalization. Further studies are needed to compare topical efficacy to other transdermal delivery methods and skin permeation enhancers. Comparative studies between ibuprofen and other topical NSAIDs (e.g., diclofenac, ketoprofen) are also necessary to determine the optimal pharmaceutical choice for acute soft tissue and articular injury in athletes.
